# Forecasting malaria dynamics based on causal relations between control interventions, climatic factors, and disease incidence in western Kenya

**DOI:** 10.7189/jogh.14.04208

**Published:** 2024-10-11

**Authors:** Bryan O Nyawanda, Simon Kariuki, Sammy Khagayi, Godfrey Bigogo, Ina Danquah, Stephen Munga, Penelope Vounatsou

**Affiliations:** 1 Kenya Medical Research Institute – Centre for Global Health Research, Kisumu, Kenya; 2 Swiss Tropical and Public Health Institute, Basel, Switzerland; 3 University of Basel, Basel, Switzerland; 4 Center for Development Research, University of Bonn, Bonn, Germany

## Abstract

**Background:**

Malaria remains one of the deadliest diseases worldwide, especially among young children in sub-Saharan Africa. Predictive models are necessary for effective planning and resource allocation; however, statistical models suffer from association pitfalls. In this study, we used empirical dynamic modelling (EDM) to investigate causal links between climatic factors and intervention coverage with malaria for short-term forecasting.

**Methods:**

Based on data spanning the period from 2008 to 2022, we used convergent cross-mapping (CCM) to identify suitable lags for climatic drivers and investigate their effects, interaction strength, and suitability ranges on malaria incidence. Monthly malaria cases were collected at St. Elizabeth Lwak Mission Hospital. Intervention coverage and population movement data were obtained from household surveys in Asembo, western Kenya. Daytime land surface temperature (LSTD), rainfall, relative humidity (RH), wind speed, solar radiation, crop cover, and surface water coverage were extracted from remote sensing sources. Short-term forecasting of malaria incidence was performed using state-space reconstruction.

**Results:**

We observed causal links between climatic drivers, bed net use, and malaria incidence. LSTD lagged over the previous month; rainfall and RH lagged over the previous two months; and wind speed in the current month had the highest predictive skills. Increases in LSTD, wind speed, and bed net use negatively affected incidence, while increases in rainfall and humidity had positive effects. Interaction strengths were more pronounced at temperature, rainfall, RH, wind speed, and bed net coverage ranges of 30–35°C, 30–120 mm, 67–80%, 0.5–0.7 m/s, and above 90%, respectively. Temperature and rainfall exceeding 35°C and 180 mm, respectively, had a greater negative effect. We also observed good short-term predictive performance using the multivariable forecasting model (Pearson correlation coefficient = 0.85, root mean square error = 0.15).

**Conclusions:**

Our findings demonstrate the utility of CCM in establishing causal linkages between malaria incidence and both climatic and non-climatic drivers. By identifying these causal links and suitability ranges, we provide valuable information for modelling the impact of future climate scenarios.

The substantial decline observed in the global trends of malaria incidence since the year 2000, largely attributable to the scale-up of malaria control interventions, has been stagnating since 2020, with the burden remaining disproportionately high in sub-Saharan Africa [1]. Changes in climatic factors threaten to expand the transmission areas and further reverse the gains made in reducing the disease burden, given that environmental and climatic factors, especially rainfall, temperature, and humidity, play a significant role in malaria dynamics [2]. The relationship between climatic factors and malaria incidence is context-specific and may vary from one geographical area to the next. Previously, we found that an increase in rainfall lagged over two months was associated with an increase in malaria incidence, while an increase in temperature lagged over one month was associated with a decrease in malaria incidence in western Kenya, with both having equal but opposing effects [3]. Additionally, we found that bed net use significantly contributed to the observed decline in malaria incidence in this area [3].

Studies conducted in various regions, including western Kenya [3–6], the Rift Valley in Kenya [7], Sri Lanka [8], and Uganda [9], have found that rainfall, lagged by a certain period, is associated with an increase in malaria incidence and mortality. Excess rainfall, on the other hand, was shown to be associated with reduced incidence of malaria because it disrupts the mosquito reproduction cycle by flushing mosquito larvae from breeding sites [8,10]. Temperature plays a significant role as well, with some studies reporting a decline in malaria incidence [3,6,9] and others reporting an increase [10,11], depending on the geographical area. Moreover, the relationship between temperature and mosquito development is seemingly nonlinear, whereby extremely low and high temperatures inhibit adult mosquitoes from thriving [12]. Ideal mosquito development occurs within a temperature range of 17–34°C [2,13].

Relative humidity, which indicates the amount of moisture in the air given a specific temperature, has also been linked to malaria incidence, with varying results [14,15]. A relative humidity of at least 60% enhances mosquitoes’ ability to bite and infect [15]. In their systematic review [15], Rahmani and colleagues found that humidity was a significant driver in 7 out of 14 studies reporting the association between humidity and malaria in Southeast Asia. Though most of these studies reported a positive association, one conversely reported a negative association, which was attributed to very high humidity levels (83–99%) [15]. In malaria-endemic areas of sub-Saharan Africa, including Kenya, an increase in humidity has been associated with an increase in malaria incidence [16].

Although other variables apart from rainfall, temperature, and relative humidity, such as solar radiation, wind speed, urbanisation, changes in land use, population movement, and health care access, also influence changes in malaria incidence at local levels, most modelling studies did not investigate their role in this sense [17]. Climate projections, for example, have shown that both rainfall and temperature will increase in western Kenya [3]. Due to the balancing but opposing effects of rainfall and temperature, it may be difficult to predict future trends in malaria incidence. Additionally, the identification of optimal lags and suitability ranges of climatic variables is important for meaningful forecasting. Previous studies have investigated the effects of climatic and non-climatic factors on malaria incidence using statistical regression models [3,9,15], which limit inference to associations, meaning they do not imply causation [18]. Moreover, due to the nonlinear relationship between climatic factors and malaria incidence, statistical models may not be the best choice for investigating causal links to enable accurate forecasting. The inability of statistical models to show causal links has often been criticised given the linearity assumption, which makes it difficult to adopt statistical modelling approaches in the development of early warning systems.

The Granger method, first proposed in the 1960s, is a commonly used method for testing causality [19]. While this method has been widely applied to nonlinear time series data in other fields [20–22], it remains underutilised in establishing causality between climatic conditions and malaria incidence. Frameworks such as empirical dynamic modelling (EDM), and particularly convergent cross-mapping (CCM), were developed to overcome limitations of Granger causality, such as the assumptions of stationarity and separability of effects. These frameworks allow researchers to test the relationships between outcome variables and nonlinear predictors [23,24]. CCM, as a data-driven approach, assumes that variables are causally linked if they are part of a coupled dynamical system determined through state space reconstruction [18,23]. Additionally, CCM enables us to establish the directionality of relationships and visualise the transmission suitability ranges, which are useful for forecasting.

The only available study employing CCM to investigate the influence of climatic factors on malaria incidence was conducted in Argentina [18]. It examined the influence of temperature and humidity on malaria incidence, but excluded rainfall and other climatic factors. It also did not consider interventions in the analysis. Given that the influence of climatic factors varies from one geographic location to another, developing methods that can be applied to local climatic, non-climatic, and incidence data are crucial for decision-makers. CCM facilitates this process as it does not require model equations or the assumptions that accompany statistical models. With this in mind, we used CCM to investigate the causal links between malaria incidence and an expanded list of climatic and non-climatic factors, and further applied EDM to make short-term forecasts of malaria incidence in western Kenya.

## METHODS

### Study area

The Kenya Medical Research Institute (KEMRI), in collaboration with the US Centers for Disease Control and Prevention (CDC), has been conducting population-based infectious disease surveillance (PBIDS) within the Health and Demographic Surveillance System (HDSS) in western Kenya since 2005. This surveillance follows approximately 30 000 people residing in 33 villages within a 5 kilometre radius from St. Elizabeth Lwak Mission Hospital (also referred to as Lwak Mission Hospital (LMH)) in a rural setting in Asembo, Siaya County. The characteristics of this population have been described previously [3,25,26].

### Data sources

#### Malaria incidence data

Children aged less than five years visiting LMH with symptoms of febrile illness (axillary temperature ≥38°C or history of fever within the past 24 hours) were tested for malaria by microscopy between 2008 and 2022. All children who tested positive for malaria were treated using artemisinin-based combination therapy (ACT) as per the national treatment guidelines. Monthly malaria incidence was estimated by dividing the monthly number of new malaria cases by the total monthly person-time of follow-up in years (person-years). Since children aged less than six months are protected against malaria through maternal antibodies, they were excluded from this analysis [27]. The proportion of the population moving into the HDSS was used as an indicator of population movement.

#### Bed net use data

We utilised household visit data collected biweekly from January 2008 to April 2015 [25] to estimate bed net use. The frequency of household visits was reduced to biannual visits thereafter, but the data collection instrument remained the same, with data collection occurring throughout the year. During the household visits, caretakers were asked if their children slept under bed nets the night preceding the visit. We aggregated data by month and used it to estimate the proportion of individuals reporting bed net use. Bed net use was the sole control intervention considered in this analysis due to the universal use of ACT and the unavailability of other control interventions such as indoor residual spraying in this area.

#### Climatic and environmental data

We included daytime land surface temperature (LSTD), rainfall, relative humidity (RH), wind speed, solar radiation, crop cover, and surface water coverage. LSTD data with a 1 × 1 km^2^ spatial and eight-day temporal resolution were extracted from the Moderate Resolution Imaging Spectroradiometer onboard NASA's Terra and Aqua satellites [28]. Downscaled rainfall data were obtained from the Climate Hazards Group InfraRED Precipitation with Station data at 1 × 1 km^2^ spatial and monthly temporal resolutions [29], while RH data were calculated from ERA5 data sets [30] using the August-Roche-Magnus formula which uses 2 m temperature (T), 2 m dew point temperature (TD), coefficient *C_a_* equal to 17.625, coefficient *C_b_* equal to 243.05 and *K_zero_* equal to 273.15 K (equivalent to 0°C) [31]. Daily wind speed and solar radiation data were extracted from ERA-5 Land daily aggregate dataset [30], while land cover and surface water coverage data were obtained from Copernicus and the Joint Research Centre (JRC) at 100 × 100 m^2^ and 30 × 30 m^2^, respectively [32,33]. Monthly averages of LSTD, rainfall and RH, wind speed, solar radiation, and surface water coverage were calculated at their original scales and then averaged within the area to allow linkage to the malaria incidence data. Crop cover in the study area did not change during the study period (Figure S1 in the **Online Supplementary Document**), while a time series of health care access – measured using time to the nearest health facility could not be generated for inclusion in this type of analysis. These two variables were therefore not considered in the analysis.

### Statistical analyses

We managed and analysed all data in R, version 4.1.3 (R Core Team, Vienna, Austria). Pearson correlation and time series plots were used for descriptive analysis. All EDM analyses were performed using the ‘rEDM’ version 1.14.0 package [34]. 

#### Convergent cross mapping

The CCM method is used in nonlinear time series analysis, particularly in EDM, to infer causal relationships between variables in a complex system [23]. Developed within the framework of nonlinear state-space reconstruction, it aims to determine whether one time series can predict another, offering insights into potential causal links.

The method uses Takens’ theorem [35] to transform an original time series into a higher-dimensional space by applying time delay embedding. This technique involves selecting an embedding dimension, which determines how many past values of the time series to use for creating a new space, known as state-space reconstruction [23]. The key feature of CCM is using the reconstructed state-space of one variable (referred to as a shadow manifold) to predict another, as described by Sugihara et al. [23]. Briefly, if variable *X* (such as climatic factors) can predict a variable *Y* (such as malaria incidence), it suggests that *X* may have a causal influence on *Y*. This is assessed by comparing the Euclidean distances between *Y’s* observed values and the predicted values derived from *X*’s reconstructed state-space. Here, CCM measures how well the predictions of the two linked variables, *X* and *Y*, align by using their shadow manifolds. It allows us to use the shadow manifold *M_y_* of *Y* to predict the statesof *X* in *M_x_*. The prediction skill (*ρ*) is the correlation coefficient between predicted and observed states of *X*, and it should improve as the length of the time series increases.

Malaria transmission is often seasonal, reflecting the seasonality of climatic factors. It is important to distinguish the effects of these drivers from their mutual seasonality to clarify causal links. After selecting the embedding dimension, which indicates the appropriate time-delay in the effect of a climatic factor on malaria [36], we separated the influence of malaria seasonality from the influence of the climatic factor using a surrogate time series for the climatic factor as previously described [18,37]. Briefly, using the observed and surrogate time series, we tested the hypothesis of no causal link between malaria and the climatic driver variables. For each driver variable *x*(*t*), a seasonal pattern *x_s_*(*t*) was obtained using smoothing splines. Residuals were then estimated by subtracting the seasonal cycle values from the observed values, i.e. *x_r_*(*t*) = *x*(*t*) − *x_s_*(*t*). These residuals were shuffled and added back to the seasonal pattern to generate a surrogate series *x̂*(*t*). If *x*(*t*) is causally linked to *y*(*t*), then *y*(*t*) should predict *x*(*t*) better than *x̂*(t), indicating that *y*(*t*) is sensitive to both the seasonal pattern and the residuals of *x*(*t*). To test this hypothesis, we calculated the *ρ *of 1000 surrogates and compared the probability distribution of the *ρ_s_* to the prediction skill of the original series. Statistical significance was considered at *α* = 0.05. All malaria driver variables were standardised to remove measurement bias and allow direct comparison of their effects.

#### Estimation of interactions’ strength and direction

After establishing causal links and the most suitable lags of the climatic factors, we investigated the strength and directionality of their effects on malaria incidence among children under five years of age. For this, we used a multivariable state space reconstruction approach which included LSTD, rainfall, RH, wind speed, in-migration and bed net use. From the reconstruction, we obtained a coefficient *C* considered a proxy for the interaction strength between each driver and malaria incidence [38]. Assuming that malaria incidence *y*(*t*) is affected by *E* different predictor variables *x_i_*,*i* = 1,2,… *E*, the state space at time *t* is given by *x*(*t*) = *x_1_*(*t*), *X_2_*(*t*),… *x_E_*(*t*), and a linear model *C* that predicts the value *y*(*t*) from the multivariable reconstructed state-space vector *X*(*t*) is given by the formula



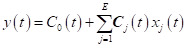



Where *C* contains the coefficients *C*_0_, *C*_1_,… *C_E_* obtained from a singular value decomposition solution as described by Ushio and Laneri [18,38].

#### Forecasting

We assessed the predictive power of individual and combined drivers at forecasted time points of six, three, and one year using univariable and multivariable simplex projection [34,39]. We fitted two forecasting models: one utilising the entire time series (2008–22), and another covering 2008–19 to eliminate potential bias related to changes in healthcare-seeking behaviour during the COVID-19 pandemic. For each forecasting time point, we utilised a subset of the data as the training set; for example, for the three-year forecasting model, we used nine years of data (from 2008–16) to predict the incidence in the following three years (from 2017–19). We used Pearson correlation, mean absolute error (MAE), and root mean square error (RMSE) to compare the observed vs predicted incidences.

## RESULTS

**Figure 1:**
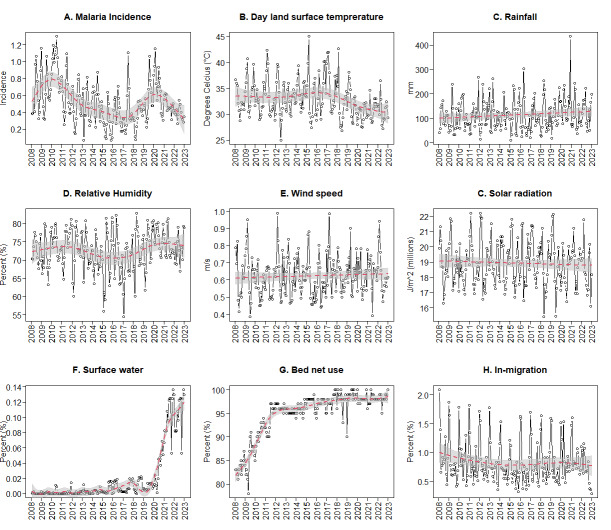
Monthly values. **Panel A.** Malaria incidence. **Panel B.** Daytime LST. **Panel C.** Rainfall. **Panel D.** Relative humidity. **Panel E.** Wind speed. **Panel F.** Solar radiation. **Panel G.** Surface water. **Panel C.** Bed net use. **Panel C.** In-migration.

Malaria incidence peaked during May to July every year in this study area (**Figure 1**), with a second smaller peak occurring from November to January. The monthly median incidence was 0.46 (interquartile range (IQR) = 0.33–0.66) cases per person-year. The median LSTD, rainfall, RH, wind speed, solar radiation, and surface water coverage were 32.1°C (IQR = 30.2–34.8°C), 101 mm (IQR = 67–152 mm), 73% (IQR = 69–77%), 0.6 m/s (IQR = 0.5–0.7 m/s), 18 884 151 J/m^2^ (IQR = 17 759 044–19 927 002 J/m^2^), and 0.003% (IQR = 0.000–0.016%), respectively. All these factors showed clear seasonal patterns. Rainfall peaked from March through May, when temperature was observed to be low (**Figure 1**).

Upon fitting splines, we observed that malaria incidence peaked in both 2009–10 and late 2019 to early 2020 (**Figure 1**). Malaria cases steadily declined until 2016, followed by a resurgence. LSTD, in turn, increased between 2012 and 2016, while average RH decreased, and average rainfall steadily increased. There was also a slight increase in wind speed, a slight decline in solar radiation and an increase in surface water coverage. Bed net use increased, while the proportion of in-migrants declined over time. In-migration was seasonal, with the highest levels occurring in December and January.

### Causal links

**Figure 2:**
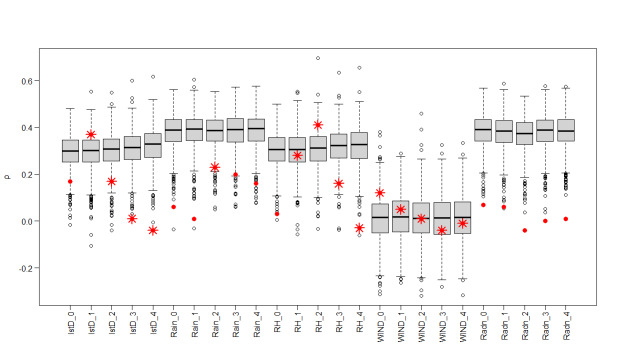
Causal relationships of daytime LST, rainfall, RH, wind speed, and solar radiations as a function of lags 0, 1, 2, 3, and 4 as determined by CCM. Boxplots present the distribution of surrogate time series. Asterisks represent time-lag values that provided a larger than expected ρ by a common seasonal trend and considered statistically significant at α = 0.05 significance level.

Using Pearson correlation analysis, we observed that LSTD lagged over the previous month (*ρ* = −0.49), rainfall lagged over the previous two months (*ρ* = 0.42), RH lagged over the two previous months (*ρ* = 0.59), wind speed in the current month (ρ = −0.31), and solar radiation in the current and previous month (*ρ* = −0.32) had the highest correlations with malaria incidence (**Table 1**). The causal relationships between these drivers and their lags, with malaria incidence were also confirmed using CCM. Specifically, LSTD lagged over the previous month, rainfall lagged over the previous two months, RH lagged over the previous two months, and wind speed in the current month exhibited the highest prediction skills (**Figure 2**).

**Table 1:** Pearson correlation coefficients between lagged climatic variables and malaria incidence

	Pearson correlation for each lag									
	**Lag 0**		**Lag 1**		**Lag 2**		**Lag 3**		**Lag 4**	
**Variable**	**ρ**	***P*-value**	**ρ**	***P*-value**	**ρ**	***P*-value**	**ρ**	***P*-value**	**ρ**	***P*-value**
LSTD in °C	−0.35	<0.001	−0.49	<0.001	−0.29	<0.001	0.10	0.192	0.28	<0.001
Rainfall in mm	−0.18	0.017	0.20	0.008	0.42	<0.001	0.29	<0.001	0.00	0.969
Relative humidity in %	0.13	0.075	0.50	<0.001	0.59	<0.001	0.26	<0.001	−0.10	0.198
Wind in m/s	−0.31	<0.001	−0.27	<0.001	0.03	0.716	0.23	0.002	0.04	0.624
Solar radiation in J/m^2^	−0.32	<0.001	−0.33	<0.001	0.00	0.993	0.12	0.110	−0.07	0.374
Surface water	−0.13	0.076	−0.13	0.089	−0.12	0.097	−0.12	0.097	−0.13	0.072

LSTD – daytime land surface temperature

### Interactions’ strength and directionality

**Figure 3:**
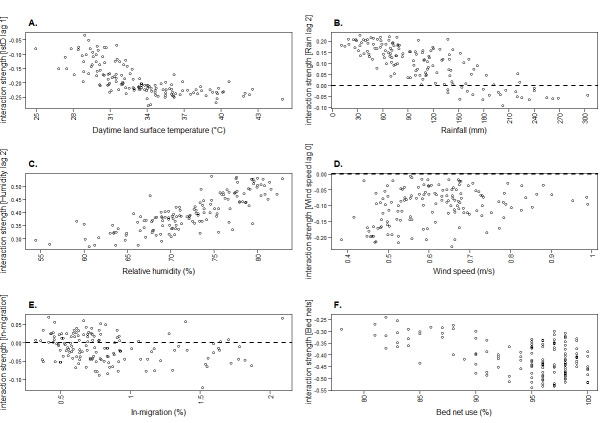
Interaction strengths for each causal variable over the respective ranges of their values. **Panel A.** Daytime LST. **Panel B.** Rainfall. **Panel C.** Relative humidity. **Panel D.** Wind speed. **Panel E.** In-migration. **Panel F.** Bed net use.

Regarding the interaction strengths and directionality for the key drivers of malaria, we found that LSTD lagged over the previous month negatively affected malaria incidence and its interaction strength increased with rising temperatures (**Table 2**, **Figure 3**, Panel A). This implies that an increase in temperature had a stronger negative effect on malaria incidence. The interaction strength of LSTD was more pronounced between 30 − 35°C.

**Table 2:** Summary of the optimal lags for the causal variables, the direction of their effect and their behaviour given different ranges

Predictor	Lag in months	Sign/effect	Range of values
LSTD in °C	1	Negative	Pronounced between 30–35°C
Rainfall in mm	2	Positive	Pronounced between 30–120 mm
Relative humidity in %	2	Positive	Pronounced between 67–80%
Wind speed	0	Negative	Pronounced between 0.5–0.7 m/s
Proportion of in-migration	NA	Not clear	<1%
Proportion of bed net use	NA	Negative	Pronounced above 95%

LSTD – daytime land surface temperature, NA – not applicable

Rainfall lagged over the previous two months, had a nonlinear interaction with malaria incidence. Overall, rainfall had a positive effect on malaria incidence and the interaction strength was more pronounced between monthly average rainfall of 30–120 mm (**Table 2**, **Figure 3****,** Panel B). However, the directionality became negative with increased monthly rainfall values, especially after 180 mm.

RH lagged over the previous two months showed a strong positive effect on malaria incidence. The interaction strength increased with higher values of relative humidity and was more pronounced between 67–80% (**Table 2**, **Figure 3**, Panel C). Wind was negatively associated with incidence, with the interaction being more pronounced between 0.5–0.7 m/s (**Table 2**, **Figure 3**, Panel D).

There was no clear trend in the association of in-migration with malaria incidence, with the interaction being highest at less than 1% in-migration (**Table 2**, **Figure 3**, Panel E). Bed net use, meanwhile, had a strong negative effect on malaria incidence (**Table 2**, **Figure 3**, Panel F), whereby the interaction strength increased with a higher proportion of children sleeping under bed nets and was more pronounced when coverage was above 95%.

### Forecasting

**Figure 4:**
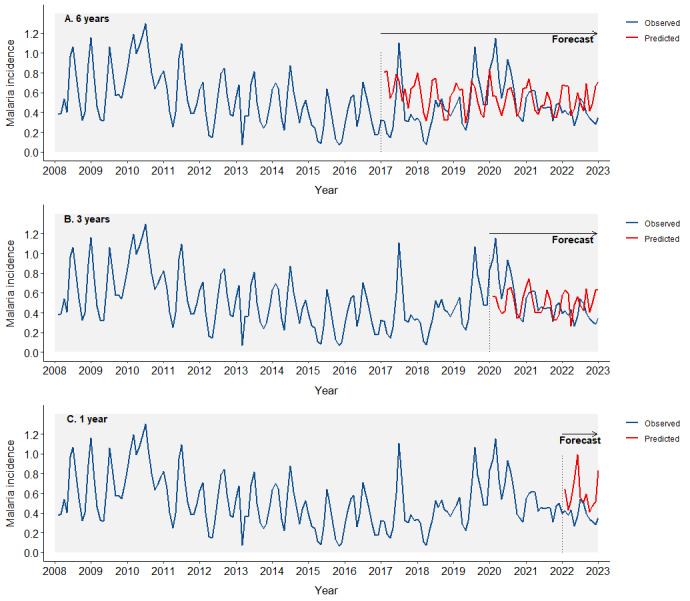
Forecasting of malaria incidence using the full data set from 2008 to 2022. Blue lines represent observed incidence, while red lines represent predicted incidence. **Panel A.** Six years. **Panel B.** Three years. **Panel C.** One year.

Using the full data set from 2009 to 2022 (**Figure 4**), we observed poor predictions of malaria incidence through the multivariable state space reconstruction, which included rainfall, LSTD, RH, and bed net use. The six-year predicted performance had a *ρ* = 0.18, R^2^ = 0.04, and RMSE = 0.25; the three-year predicted performance achieved a *ρ* = 0.21, R^2^ = 0.04, and a RMSE = 0.21; and the 1-year predicted performance estimated a *ρ* = −0.14, R^2^ = 0.02, and a RMSE = 0.29 (**Table 3**).

**Table 3:** Assessment of forecasting accuracy for different lead times using the full 2008–22 data set and the 2008–19 subset

	Forecasting period		
**Analysis period and accuracy measure**	**Six years**	**Three years**	**One year**
2008**–**22			
*Rho (ρ)*	0.18	0.21	−0.13
*R^2^*	0.03	0.04	0.02
*MAE*	0.21	0.17	0.22
*RMSE*	0.25	0.21	0.29
2008**–**19			
*Rho (ρ)*	0.49	0.46	0.85
*R^2^*	0.24	0.21	0.73
*MAE*	0.26	0.21	0.12
*RMSE*	0.30	0.24	0.15

MAE – mean absolute error, RMSE – root mean square error

**Figure 5:**
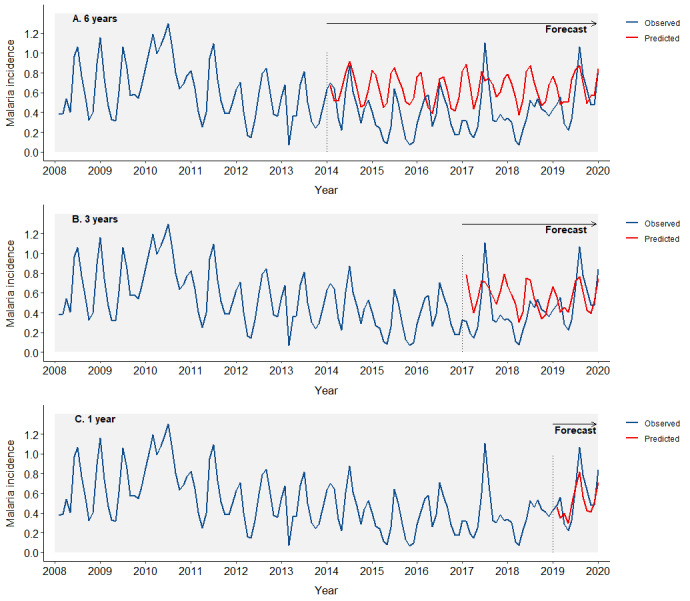
Forecasting of malaria incidence using the data set from 2008 to 2019. Blue lines represent observed incidence, while red lines represent predicted incidence. **Panel A.** Six years. **Panel B.** Three years. **Panel C.** One year.

When utilising the 2008–19 (pre-COVID-19) time series in forecasting (**Figure 5**), we observed a significant improvement in predictive ability. The six-year forecasts had a *ρ* = 0.49, R^2^ = 0.24, and RMSE = 0.30; the three-year forecasts had a *ρ* = 0.46, R^2^ = 0.21, and RMSE = 0.24; and the one-year forecast had a *ρ* = 0.85, R^2^ = 0.73, and RMSE = 0.15 (**Table 3**).

Though the predictive ability of all the drivers was not clear when the full-time series was used (Figure S2 in the **Online Supplementary Document**), we observed good predictive performance for LSTD (*ρ* = 0.87, R^2^ = 0.76, RMSE = 0.15) and rainfall (*ρ* = 0.84, R^2^ = 0.71, RMSE = 0.16) using the pre-COVID-19 data set (Figure S3 in the **Online Supplementary Document**). We also found that malaria incidence from earlier time points was a good predictor of future incidence (*ρ* = 0.86, R^2^ = 0.74, RMSE = 0.20).

## DISCUSSION

Using CCM, we observed causal links between LSTD lagged over the previous month, rainfall and RH lagged over the previous two months; wind speed in the current month; and bed net use with malaria incidence among children under five years of age. An increase in LSTD, wind speed, and bed net use was associated with a decline in malaria incidence. Conversely, an increase in rainfall and RH was associated with an increase in malaria incidence. Rainfall exceeding 180 mm, meanwhile, was negatively associated with malaria incidence. CCM effectively captured the nonlinear relationships between climatic drivers and malaria incidence.

Our findings demonstrated the effectiveness of EDM in short-term malaria incidence forecasting for this area, showing better prediction accuracy for shorter timescales. Furthermore, we elucidated the transmission suitability ranges considering the combined impact of climatic drivers, population movement, and bed net use. These findings are essential for both short and long-term forecasting under different climate scenarios and are useful for developing climate adaptation tools, including malaria early warning systems.

We previously found a significant association between temperature and malaria incidence in the study area using statistical models, consistent with findings from other studies [3,4,6,9]. Here we confirmed a similar temperature lag over the previous month, as previously established through statistical methods [3], and provided evidence of a causal link between temperature and malaria incidence. We found that LSTD between 30–35°C was associated with increased malaria transmission, while there was a greater reduction in malaria incidence with LSTD beyond 35°C. Considering that the mean difference between LSTD and air temperature is approximately 2°C, the findings from this study align with previous studies showing that optimal temperature for development of adult mosquitoes was between 28–32°C [2,12,40]. Elsewhere, temperature ranges between 17–34°C were associated with increased malaria transmission, indicating the importance of considering the geographical context and the study type (either laboratory- or field-based) [13]. With a warming climate, and considering that malaria transmission is complex and influenced by multiple factors, including land use and topography, transmission may reduce in this area if only temperature is considered. However, it may expand to areas currently classified as low transmission areas, particularly the cooler highland regions [41–43]. CCM provides researchers with an opportunity to understand context-specific lags and suitable temperature ranges, thus allowing for the prediction of future malaria dynamics, better planning, resource allocation, and targeting of intervention tools.

An increase in rainfall, lagged over the previous two months, was causally linked to an increase in malaria incidence, particularly when rainfall levels were between 30 and 120 mm. However, excessive rainfall (above 180 mm) was negatively associated with malaria incidence. These findings align with observations from other studies, where malaria transmission peaked at rainfall levels between 80 and 120 mm [6,44]. Excessive rainfall can lead to the flushing of mosquito larvae from their habitats, reducing larval numbers and disrupting the mosquito reproduction cycle [8,10]. With climate change, certain regions may experience increased rainfall, potentially triggering epidemic malaria transmission. By identifying the optimal range of rainfall for malaria transmission, control programmes can collaborate with meteorological counterparts to predict the occurrence and magnitude of malaria incidence, allowing for advanced planning, targeting of interventions and resource allocation.

Though most studies only used temperature and rainfall as climatic drivers of malaria dynamics, several have considered relative humidity in their analysis. In our study, we included RH as a predictor and established causal links between RH and malaria incidence. We observed a steady positive relationship between the 67–80% RH range with an optimal lag for humidity being the previous two months. Previous studies have associated increased relative humidity with an increase in incidence, highlighting RH levels above 60% as optimal for mosquitoes to bite and infect [16,45]. However, it is important to note that excessive RH, like excessive rainfall, was previously found to be negatively associated with malaria incidence [15]. Additionally, we evaluated wind speed, solar radiation, and surface water coverage, and excluded changes in land cover since this did not change over time in the relatively small study area. Previous research found an increase in wind speed to be negatively associated with malaria incidence; winds alter the abundance of malaria vectors, and an increase in wind speed results in a decrease in mosquito biting rates [46–48]. Solar radiation and surface water coverage were excluded from further analysis after no significant relationship was established.

Most EDMs only evaluate climatic drivers. However, we included population movement (in-migration) and bed net use in our model to assess their causal links with changes in malaria incidence in this area. Migration is likely to increase transmission due to the importation of cases [49]. However, we did not find a clear association between in-migration and malaria incidence, likely due to the very low number of individuals moving into the HDSS area. We did find that bed net use was negatively associated with malaria incidence, consistent with previous studies [3,4,50,51]. Bed net coverage greater than 90% was linked with the greatest reduction in incidence. With climate projections indicating an increase in both temperature and rainfall, we believe that, if bed net use is optimised and over 90% of residents are covered, there will be a significant reduction in malaria incidence in this area in the coming years. This finding underscores the importance of bed net use in malaria control.

The ability of EDM to predict short-term malaria incidence provides an alternative methodology for early warning systems that navigate the pitfalls of statistical models, especially with good quality long time series data. With good quality data collected during 2008–19, EDM was able to predict malaria incidence well, yet it displayed poor predictive ability when the full data set covering the COVID-19 pandemic period was used. This may be due to disruptions in healthcare-seeking during the COVID-19 pandemic period, which resulted in lower healthcare-seeking post-2019. A recent analysis comparing healthcare-seeking behaviour before and during the pandemic reported a 19% decline in the odds of seeking health care at a health facility due to the COVID-19 pandemic in the study area [52]. Malaria stakeholders, especially the malaria control programme and other players like county health departments in Kenya, can employ this straightforward method to forecast malaria activity for planning purposes in both endemic and epidemic zones. Furthermore, it allows for the inclusion of control intervention coverage, enabling informed decisions about coverage thresholds and the estimation of when malaria elimination can be achieved. By using climate and control intervention suitability ranges, one can estimate malaria incidence over prolonged periods given different climate scenarios, which may help in projecting realistic elimination timelines.

Lastly, CCM, like the frequentist and Bayesian statistical models, can be used for variable selection and determining the most appropriate lags for climatic drivers. However, unlike statistical models, which are limited to assessing associations only, it allows for the evaluation of causal links between malaria incidence and climatic and non-climatic time series drivers. Its main limitation is that it may not be able to distinguish between bidirectional causality and strong unidirectional causality that results in synchrony [36]. However, this is not a concern for this study, as we assume that the relationship between climatic drivers and disease incidence is unidirectional. Another limitation is the consideration of bed net use as the only malaria control tool in the study area due to the unavailability of data on other interventions. While this study did not account for ACTs, as all cases testing positive for malaria were treated with ACTs, future studies with varied coverage in ACT use or other control interventions should consider incorporating the coverage of these interventions in their analysis. Lastly, since there were no weather stations in the area, we used downscaled and reanalysed climatic data from remote sensing for this analysis, which may not be very accurate. Future analyses should validate remotely sensed data against weather stations’ data as they become available.

## CONCLUSIONS

Our findings show the utility of CCM in establishing causal linkage between malaria incidence and both climatic and non-climatic drivers. They can also aid the selection of optimal time lags for climatic drivers and the identification of interaction strengths and suitability ranges of these factors. We also showed the utility of space-time reconstruction in short-term forecasting. The causal links and suitability ranges provided here can be valuable for modelling the impact of future climate scenarios, and these methods can be applied in other areas for the evaluation of area-specific links and suitability ranges.

## Additional material


Online Supplementary Document
