# Supplementary material for: Forecasting malaria dynamics based on causal relations between control interventions, climatic factors, and disease incidence in western Kenya
Source: J Glob Health. 2024 Oct 11;14:04208. doi: 10.7189/jogh.14.04208 (PMC11466501; doi:10.7189/jogh.14.04208)
Supplement: Online Supplementary Document [file jogh-14-04208-s001.pdf]

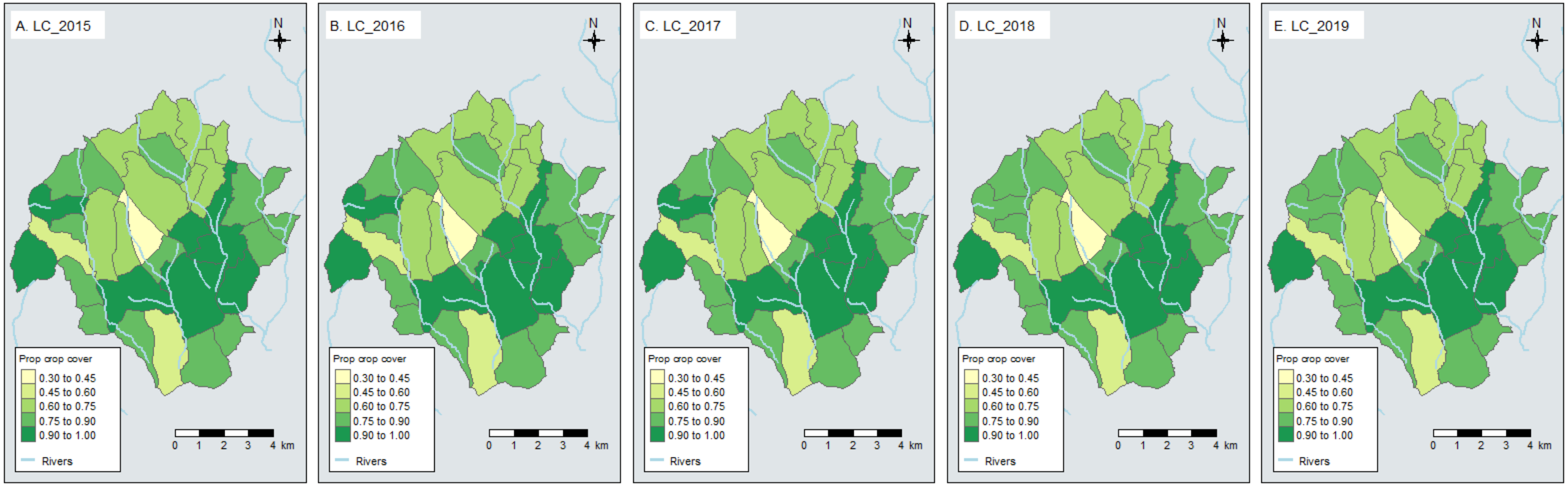

Figure S1: The annual distribution of crop cover in the study area from 2015-2019 indicating no change over this period.

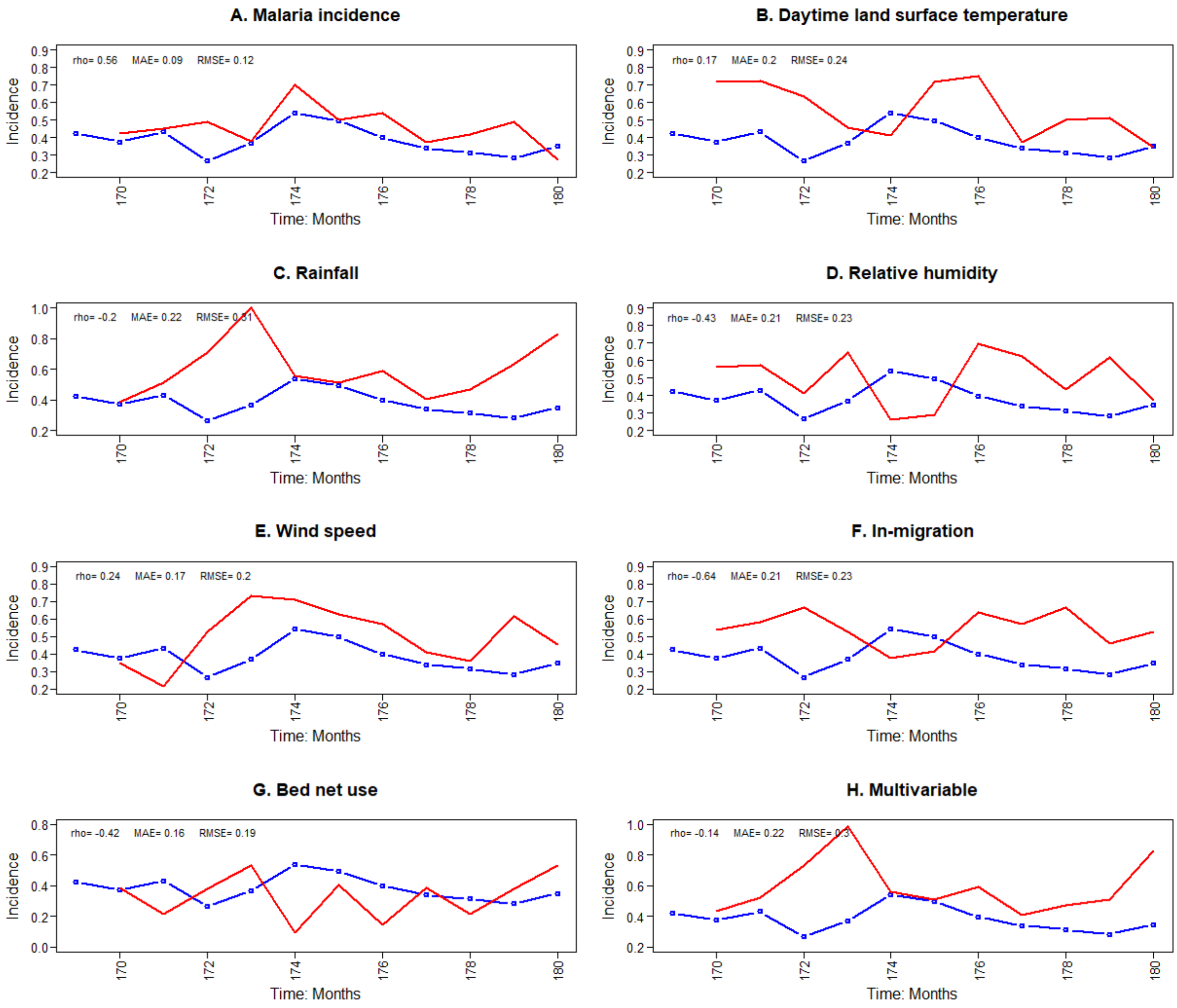

Figure S2: One year forecasting of monthly malaria incidence using (A) malaria incidence; (B) daytime land surface temperature LSTD; (C) rainfall; (D) relative humidity; (E) wind speed; (F) in-migration; (G) bed net use; and (H) the six predictors together. Data is analysed from 2008 to 2022. Blue lines represent observed incidence, while red lines represent predicted incidence.

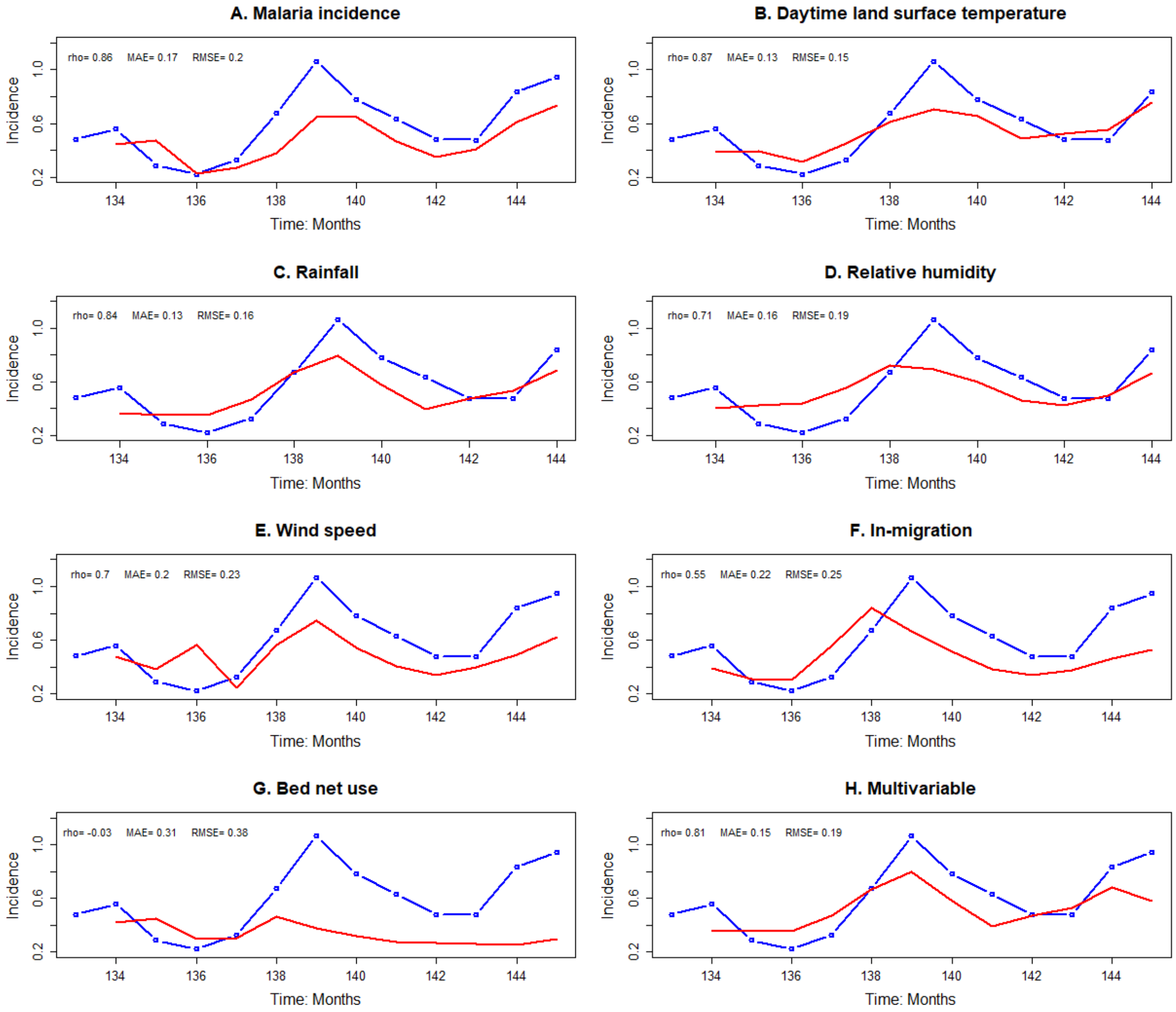

Figure S3: One year forecasting of monthly malaria incidence using A) malaria incidence; (B) daytime land surface temperature LSTD; (C) rainfall; (D) relative humidity; (E) wind speed; (F) in-migration; (G) bed net use; and (H) the six predictors together. Data is analysed from 2008 to 2019. Blue lines represent observed incidence, while red lines represent predicted incidence.
